# Exogenous and endogenous determinants of vitamin K status in cystic fibrosis

**DOI:** 10.1038/srep12000

**Published:** 2015-07-10

**Authors:** Patrycja Krzyżanowska, Andrzej Pogorzelski, Wojciech Skorupa, Jerzy Moczko, Philip Grebowiec, Jarosław Walkowiak

**Affiliations:** 1 Department of Pediatric Gastroenterology and Metabolic Diseases, Poznan University of Medical Sciences, Poznan, Poland; 2 Department of Pneumology and Cystic Fibrosis, Institute of Tuberculosis and Lung Diseases, Rabka, Poland; 3 National Tuberculosis and Lung Diseases Research Institute, Warsaw, Poland; 4 Department of Informatics and Statistics, Poznan University of Medical Sciences, Poznan, Poland

## Abstract

Cystic fibrosis (CF) patients are at high risk for vitamin K deficiency. The effects of vitamin K supplementation are very ambiguous. Therefore, we aimed to define the determinants of vitamin K deficiency in a large cohort of supplemented - 146 (86.9%) and non-supplemented - 22 (13.1%) CF patients. Vitamin K status was assessed using prothrombin inducted by vitamin K absence (PIVKA-II) and undercarboxylated osteocalcin (u-OC). The pathological PIVKA-II concentration (≥2 ng/ml) and abnormal percentage of osteocalcin (≥20%) were found in 72 (42.8%) and 60 (35.7%) subjects, respectively. We found that liver involvement, diabetes, and glucocorticoid therapy were potential risk factors for vitamin K deficiency. Pathological concentrations of PIVKA-II occurred more frequently in patients with pancreatic insufficiency and those who have two severe mutations in both alleles of the *CFTR* gene. Pathological percentage of u-OC was found more frequently in adult CF patients and those not receiving vitamin K. However, it seems that there are no good predictive factors of vitamin K deficiency in CF patients in everyday clinical care. Early vitamin K supplementation in CF patients seems to be warranted. It is impossible to clearly determine the supplementation dose. Therefore, constant monitoring of vitamin K status seems to be justified.

Vitamin K belongs to the class of fat-soluble vitamins^1^. The term vitamin K includes the subtypes phylloquinone (vitamin K_1_), menaquinones (vitamin K_2_) and menadione (vitamin K_3_)^1^,^2^. These compounds have the same 2-methyl-1,4-naphthoquinone backbone structure but differ by the substituent on the third carbon of the naphthoquinone ring^1^. Vitamin K is a cofactor for the enzyme γ-glutamylcarboxylase, which supports posttranslational carboxylation of glutamic acid residues in proteins such as coagulation factors (II, VII, IX, X), proteins C, S and Z, osteocalcin, Matrix Gla Protein (MGP), Growth Arrest Specific Gene 6 (Gas-6), the Transmembrane Gla proteins (TMG3 and TMG4), the Proline-Rich Gla proteins (PRGP1 and PRP2), the GlaRich Protein (GRP), periostin and transthyretin^1^,^3^. Undercarboxylated forms of these proteins, which are functionally defective, appear in vitamin K deficiency^3^.

Prothrombin induced in vitamin K absence - II (PIVKA-II) and undercarboxylated osteocalcin (u-OC) are very sensitive markers of vitamin K deficiency. Osteocalcin is the first protein to become undercarboxylated in vitamin K deficiency, and the last to respond to supplements^4^,^5^.

Vitamin K deficiency is frequently observed in patients with cystic fibrosis (CF)^6^. Rana *et al.* observed that low vitamin K status was present in 29% CF patients less than 18 years old. Moreover, fat-soluble vitamin deficiency was present in 10%–35% of children with pancreatic insufficiency^7^. It has been suggested that the deficiency is a result of fat malabsorption, bowel resection, liver disease, and chronic antibiotic treatment^8^. The manifestation of vitamin K deficiency may include bleeding (via the nose, gastrointestinal system, and urine), bruising, and osteoporosis^9^.

Although vitamin K supplementation in CF patients seems to be necessary, the dose required to prevent deficiency is unclear^10^. Evidence from randomized controlled trials on vitamin K supplementation for CF patients is currently weak and limited to two small trials (total of 32 participants). Both trials reported normal levels of undercarboxylated osteocalcin after one month of daily supplementation with 1 mg of vitamin K^9^. Siwamogsatham *et al.* found that despite nearly doubling fat-soluble vitamin dosage (including vitamin K) in CF patients with pancreatic insufficiency over a 5 year period, there was no parallel increase in blood concentrations of these vitamins^11^.

The aims of the present study were to assess the frequency of vitamin K deficiency in CF patients and to estimate exogenous and endogenous determinants of vitamin K status.

## Results

Body weight and height two standard deviations below the mean values were found in 27 (16.1%) and 39 (23.2%) patients, respectively. Hypoalbuminemia was documented in 30 (17.9%) CF patients. Spirometry was performed in 133 (79.2%) patients older than 6. Proper lung function was observed in 46 (34.6%) patients, while significant impairment in spirometry was found in 36 (27.1%) patients. Pathological concentrations of the liver enzymes AST, ALT, and GGT were found in 29.2%, 29.2% and 20.2% of patients, respectively. Comprehensive clinical data in the study group have been presented in Table 1.

Twenty two (13.1%) patients were pancreatic sufficient, while the remaining 144 (85.7%) subjects presented with steatorrhea. Liver cirrhosis and liver involvement were documented in 9 (5.4%) and 47 (28.0%) patients, respectively. Ninety-six (57.1%) patients were colonized with *Pseudomonas aeruginosa*. Eighteen (10.7%) CF patients had diabetes.

One hundred and fifty (89.3%) subjects received vitamin K; however, 4 of these patients received a dose lower than recommended. The supplementation dose ranged from 1.05 to 70 mg/week (mean ± SD: 14.9 ± 10.9 mg/week, median: 10, 1^st^–3^rd^ quartile: 5.6–20.0). Eighteen (11.7%) patients did not receive vitamin K. One hundred forty six (86.9%) patients were supplemented with pancreatic enzymes. Forty six (27.4%) and 42 (25.0%) subjects were treated permanently with oral and inhaled antibiotics, respectively. Thirty-one (18.5%) and 100 (59.5%) individuals were treated with oral and intravenous antibiotics in the three preceding months, respectively. Inhaled glucocorticoids were taken by 81 (48.2%) patients.

Median PIVKA-II concentrations and percentages of u-OC in the study group were 1.5 ng/ml (1^st^–3^rd^ quartile: 0.5–3.4) and 10.5% (1^st^–3^rd^ quartile: 3.9–44.8). The pathological PIVKA-II concentration (≥2 ng/ml) and abnormal percentage of osteocalcin (≥20%) were found in 72 (42.8%) and 60 (35.7%) subjects studied, respectively.

A description of the clinical parameters in the subgroups of CF patients with both normal and pathological PIVKA-II concentrations and percentages of u-OC have been presented in Tables 2 and 3.

Vitamin K deficiency defined as PIVKA-II ≥ 2 ng/ml was much more frequent in patients with pancreatic insufficiency, the genotype F508del/F508del or the severe mutations in both alleles of the *CFTR* gene, and those not receiving vitamin K (Table 2).

Vitamin K deficiency based on abnormal u-OC% was found more frequently in adults CF patients and those not receiving vitamin K (16<76.2%> vs. 44<30.8%>, p = 0.000036; Fisher-Freeman-Halton test) (Table 3)

A multiple regression analysis showed that none of the independent variables were important for predicting PIVKA-II or u-OC status (independent variables in the regression model: age, Z-score for body weight and height, FEV1, albumin concentration, vitamin K dose mg/week, diabetes, liver disease, pancreatic insufficiency, *Pseudomonas aeruginosa* colonization, inhaled and oral permanent antibiotic therapy, intravenous and oral antibiotic therapy in the preceding three months, inhaled glucocorticoid therapy, *CFTR* mutation). In a stepwise multiple regression, the independent variables of liver disease, inhaled glucocorticoid therapy, diabetes and *Pseudomonas aeruginosa* colonization were entered according to their statistical contribution in explaining the variance of PIVKA-II. Similarly, vitamin K dose, inhaled glucocorticoid therapy, and intravenous antibiotic therapy in the preceding three months were added to the regression equation according to their statistical contribution in explaining the variance of u-OC. The results of the stepwise multiple regression models have been summarized in Table 4.

## Discussion

The main possible causes of vitamin K deficiency in CF patients include fat malabsorption, liver disease, chronic antibiotic treatment, bowel resection, and increased mucous accumulation in the small intestine. Very often, CF patients do not have proper vitamin K status despite vitamin K supplementation^8^,^10^,^12^. Therefore, we aimed to define the exogenous and endogenous determinants of vitamin K deficiency in CF patients in a large cohort of supplemented and non-supplemented CF patients.

Vitamin K deficiency was characterized in the past as prolonged prothrombin time (PT) and activated partial thromboplastin time (APTT), as well as low concentrations of vitamin K-dependent coagulation proteins such as factors II, VII, IX and X^13^. However, coagulopathy is an insensitive marker of vitamin K deficiency because it may appear when the amount of undercaboxylated coagulation factors exceeds 50%^4^. PIVKA-II may be detectable before any changes in coagulation tests appear^13^. Therefore, this parameter seems to be a sensitive marker for early vitamin K deficiency^12^,^14^. In the present study, subjects with abnormal PIVKA-II levels had higher INR values compared to patients with a normal vitamin K status (p = 0.0178). However, only 4 (5.6%) out of the 72 CF patients with pathological PIVKA-II concentrations were found to have abnormal values of PT% (data not shown) and INR.

In the present study, pathological PIVKA-II concentrations were found in 38.4% of CF patients receiving and 72.7% of those not receiving or receiving less than the recommended dosage of vitamin K for CF. Based on the percentage of u-OC, an insufficiency (u-OC 20–50%) and a deficiency (u-OC > 50%) of vitamin K were observed in 13.3% and 17.5% vitamin K supplemented subjects, respectively. In turn, vitamin K insufficiency was observed in 14.3% and deficiency in 61.9% of patients without supplementation. The previous studies have yielded similar results, although the applied doses of vitamin K supplementation were different (≤86 μg/d - 10 mg/d)^6^,^8^,^15^,^16^,^17^. Therefore, the use of the typical recommended vitamin K dose (2.1–10 mg/week) in CF patients^18^, may not be sufficient to maintain a normal vitamin K status.

The distribution of vitamin K deficiency or insufficiency and normal vitamin K status was different for adults and children. However, a multiple regression analysis showed that age was not a significant predictor of vitamin K status. Dougherty *et al.* found that the age of males was a significant predictor of u-OC percentage through a multiple regression analysis^6^.

Fat and bile malabsorption were suggested to be the main causes of fat-soluble vitamin deficiency in CF^19^. CF subjects with pancreatic insufficiency were proven to have significantly higher PIVKA-II concentrations than pancreatic sufficient patients or healthy controls^14^,^20^. Furthermore, Nicolaidou *et al.* documented higher levels of u-OC in CF subjects with pancreatic insufficiency than in healthy subjects^17^. In the present study, pathological PIVKA-II concentrations appeared more frequently in patients with the genotype F508del/F508del or with two severe mutations in both alleles. Unfortunately, there are no published data concerning the relationship between the *CFTR* gene mutations and vitamin K deficiency. However, severe mutations such as F508del are generally associated with pancreatic insufficiency and a more severe course of the disease^21^.

Based on a stepwise multiple regression analysis, liver disease, diabetes and glucocorticoid therapy were potentially defined as determinants of PIVKA-II concentrations, and the dose of vitamin K was for u-OC percentage. However, the multiple regression analysis failed to show any statistical significance in any of the regression models. Therefore none of the independent variables seem to be a strong predictive risk factor of vitamin K deficiency in CF.

Generally, vitamin K deficiency is common in patients with cholestatic and noncholestatic liver disease. Liver disease impairs γ-carboxylation of vitamin K-dependent proteins such as PIVKA-II^14^,^22^. Rashid *et al.* showed that all CF subjects with liver disease had higher levels of PIVKA-II than patients without liver involvement^14^. Additionally, Wilson *et al.* found abnormal PIVKA-II concentrations in all CF subjects with liver disease^16^.

There is no data concerning the potential influence of diabetes on vitamin K status in CF patients. However, some of the available evidence shows that vitamin K may decrease the risk of diabetes in healthy people^23^,^24^. Similarly, there is no evidence that glucocorticoid therapy affects vitamin K status in CF. In the general population, Mokuda *et al.* showed that oral glucocorticoid dosage is an independent risk factor of lowering u-OC in post-menopausal women^25^.

To summarize, although we documented some statistical relationships and differences, there have been no strong predictive factors of vitamin K deficiency in CF patients in the present study. Overall, the data concerning this issue is limited. Dougherty *et al.* found that vitamin K supplementation (p = 0.006), 25(OH)D_3_ (p = 0.007), age for males (p = 0.045), and vitamin K supplementation only for females (p = 0.001) were significant predictors of % u-OC^6^. Nicolaidou *et al.* showed that vitamin K supplementation was a predictor of u-OC (p < 0.001), c-OC (p = 0.013), carboxy-terminal propeptide of type I procollagen (p < 0.001), and amino-terminal propeptide of type I procollagen (p < 0.001) concentrations in CF subjects^17^. The biochemical indices of bone turnover (carboxy-terminal propeptide of type I procollagen, amino-terminal propeptide of type I procollagen - markers of early osteoblastic function, carboxy-terminal telopeptide of type I collagen - marker of bone resorption) and bone mineral density (BMD) determined by dual-energy X-ray absorptiometry (DEXA) are utilized in investigating impact on bone health of vitamin K which suggests an increasingly important role of vitamin K in bone health^5^,^17^.

Available evidence also suggests that vitamin K is involved in inflammatory responses associated with bone metabolism. Kim *et al.* found that serum hs-CRP concentrations were positively correlated with vitamin K deficiency. The authors suggested that vitamin K influences the inflammatory status and bone formation^26^. Shea *et al.* found that proinflammatory cytokines such as IL-6, intercellular adhesion molecule-1 (ICAM-1), and tumor necrosis factor receptor 2 (TNFR2) were negatively correlated with vitamin K and hs-CRP levels^27^,^28^. This may be important to consider in further studies.

The major limitation of the present study is the lack of a direct measure of vitamin K status. Both PIVKA-II concentration and u-OC percentage are indirect measures, and may not ideally reflect vitamin K_1_ and K_2_ concentrations. Another limitation is a lack of vitamin K dietary intake assessment. However, the supplementation doses are many times higher, potentially reducing the significance of differences between individual dietary intakes. The large and well defined clinical study group is the main strength of this study.

In conclusion, vitamin K deficiency in CF patients seems to be very common and persists despite its supplementation. Liver involvement, diabetes, and glucocorticoid therapy were documented to be potential risk factors. Pathological concentrations of PIVKA-II are found more frequently in patients with pancreatic insufficiency and those who have two severe mutations in both alleles of the *CFTR* gene. However, it seems that there are no good predictive factors of vitamin K deficiency in CF patients in everyday clinical care. Early vitamin K supplementation in CF patients seems to be warranted. It is impossible to clearly determine the supplementation dose. Therefore, constant monitoring of vitamin K status seems to be justified.

## Methods

The studied group comprised of 168 CF patients (78 [46.4%] females and 90 [53.6%] males aged 1.2–41.4 years; 48 adults [28.6%] and 120 children [71.4%]) who prospectively underwent vitamin K status assessement. The diagnosis was based upon generally accepted guidelines^29^.

Mutations in one or both alleles of the *CFTR* gene were identified in 152 (90.5%) patients. The *CFTR* genotype could not be determined in the remaining 16 (9.5%) patients. The genotypes of the studied patients were as follows: F508del/F508del (n = 74); F508del/- (n = 23); F508del/3849 + 10 kbC > T (n = 6); F508del/2143delT (n = 6); F508del/R553X (n = 4); F508del/2183AA > G (n = 3); F508del/1717-1G>A (n = 3); F508del/CFTRdele2,3(21 kb) (n = 3); F508del/3272-26A > G (n = 2); F508del/N1303K (n = 2); F508del/4374 + 1G > T (n = 1); F508del/621 + 1G > T (n = 1); F508del/3659delC (n = 1); F508del/G1244R (n = 1); F508del/G542X (n = 1); F508del/R117H (n = 1); F508del/R334W (n = 1); G542X/- (n = 2); CFTRdele2,3(21 kb)/- (n = 2); CFTRdele2,3(21 kb)/CFTRdele2,3(21 kb) (n = 1); 1717-1-G > A/CFTRdele2,3(21 kb) (n = 1); 3849 + 10 kbC > T/- (n = 1); 3849 + 10 kbC > T/1717 − 1A > G (n = 1); N1303K/- (n = 1); N1303K/3272-26A > G (n = 1); G542X/R553X (n = 1); 1524 + 1G > A/E585X (n = 1); 2183AA > G/- (n = 1); 2184insA/622-1G > A (n = 1); 2143delT/R1102X (n = 1); 3272-26A > G/- (n = 1); 3659delC/- (n = 1); R347P/R347P (n = 1); S1196X/Q1382X (n = 1).

The nutritional status (standardized value of the body weight and height^30^, and albumin concentration); clinical expression of the disease (lung function - spirometry, exocrine pancreatic function - fecal elastase-1^31^,^32^,^33^, biochemical markers of liver function - ALT, AST, GGT, colonization of *Pseudomonas aeruginosa*, diabetes, liver cirrhosis^34^, *CFTR* genotype); international normalized ratio (INR/ as reflectance) the treatment (supplementation of vitamin K and pancreatic enzymes, inhaled and oral permanent antibiotic therapy, intravenous and oral antibiotic therapy in the preceding three months, and inhaled glucocorticoid therapy) were assessed. Vitamin K status was estimated by PIVKA-II concentration and the percentage of u-OC. PIVKA-II concentration was assessed using the Asserachrom PIVKA-II immunoassay kit (DeCarboxy Prothrombin, Diagnostica Stago, Asnières-sur-Seine, France). The percentage of u-OC was calculated using the following formula: u-OC[ng/ml]*100%/(u-OC[ng/ml] + c-OC[ng/ml]). Concentrations of carboxylated (c-OC) and undercarboxylated (u-OC) osteocalcin were assessed using the Gla-type Osteocalcin EIA and Glu-type Osteocalcin EIA immunoassay kits: (Takara BIO INC. Otsu, Shiga, Japan).

The Shapiro-Wilk test was used to check the normality of the data distribution. The Mann-Whitney test and chi^2^-test were used to assess differences between the subgroups with normal (PIVKA-II < 2 ng/ml) and abnormal (PIVKA-II ≥ 2 ng/ml) vitamin K statuses. In turn, differences between subgroups of patients with normal (u-OC < 20%), insufficiency (u-OC 20–50%) and deficiency (u-OC > 50%) of vitamin K status were assessed using the ANOVA test (for Gaussian distribution), Kruskal Wallis test (for non-Gausssian distribution), or Fisher-Freeman-Halton test. The potential influence of all studied parameters on the occurrence of vitamin K deficiency were assessed using multiple logistic regression and stepwise multiple regression analysis. The level of significance was set at p < 0.05.

The study was conducted in accordance with the revised Declaration of Helsinki. Informed, written consent was obtained from patients’ parents and from patients who were 16 years or older. The study was approved by the Bioethical Committee at Poznan University of Medical Sciences (decision 640/2009).

## Additional Information

**How to cite this article**: Krzyżanowska, P. *et al.* Exogenous and endogenous determinants of vitamin K status in cystic fibrosis. *Sci. Rep.* **5**, 12000; doi: 10.1038/srep12000 (2015).

## Figures and Tables

**Table 1:** Clinical parameters in study group.

Parameter	Median	1^st^–3^rd^ quartile
Body weight (Z-score)	–0.94	–1.77 – – 0.33
Body height (z-score)	–0.83	–1.88 – 0.06
FEV1 [%]	67.5	37.0 – 89.2
Albumin [g/dl]	3.90	3.59 – 4.23
ALT [U/L]	25.9	19.8 – 35.2
AST [U/L]	30.8	24.2 – 41.8
GGT [U/L]	15.4	11.0 – 27.7
INR	1.06	1.01 – 1.16
Vitamin K dose [mg/week]	10.0	5.6 – 20.0
Vitamin K dose [mg/kg/week]	0.37	0.23 – 0.54
Enzyme dose [FIP/kg/day]	3708	1782 – 5192

**Table 2:** Clinical parameters in CF patients with normal and pathological PIVKA-II concentration.

PIVKA-II [ng/ml]								
Parameters Median (1^st^–3^rd^ quartile)	<2	≥2	p	Parameters (N%)		<2	≥2	p
Age [years]	14.1 (8.2–19.5)	12.7 (3.3–17.2)	0.0549	Age [years]	<18	64 (53.3)	56 (46.7)	0.1241
					≥18	32 (66.7)	16 (33.3)	
Body weight (Z-score)	−1.10 (−1.81– −0.42)	−0.90 (−1.59– −0.31)	0.6148	Body weight (Z-score)	<−1	49 (59.8)	33 (40.2)	0.5039
					>−1	47 (54.7)	39 (45.3)	
Body height (Z-score)	−0.90 (−1.88– −0.04)	−0.80 (−1.88– −0.10)	0.8075	Body height (Z-score)	<−1	47 (63.5)	27 (36.5)	0.1387
					>−1	49 (52.1)	45 (47.9)	
FEV1 [%]	67.5 (30.7–89.0)	67.3 (40.5–89.4)	0.6406	FEV1 [%]	<80%	55 (63.2)	32 (36.8)	0.7902
					>80%	28 (60.9)	18 (39.1)	
Albumin [g/dl]	3.85 (3.52–4.20)	3.90 (3.61–4.24)	0.3272	Albumin [g/dl]	<3.5	20 (66.7)	10 (33.3)	0.2448
					≥3.5	76 (55.1)	62 (44.9)	
ALT [U/l]	22.0 (16.0–32.0)	26.0 (21.0–30.0)	0.0582	*CFTR* gene mutation	F508del/F508del	36 (48.6)	38 (51.4)	0.0484
					other/other	60 (63.8)	34 (36.2)	
					s/s^1^	54 (50.5)	53 (49.5)	0.0206
					other/other	42 (68.9)	19 (31.1)	
AST [U/l]	29.2 (20.9–40.7)	33.0 (27.5–46.2)	0.0160	Liver diseases	Cirrhosis	5 (55.6)	4 (44.4)	1.0000
					Other liver involvement	27 (57.4)	20 (42.6)	
					No	64 (57.1)	48 (42.9)	
GGT [U/l]	14.0 (10.0–25.2)	13.0 (11.0–25.2)	0.9910	Diabetes	Yes	12 (66.7)	6 (33.3)	0.3875
					No	84 (56.0)	66 (44.0)	
				*Ps. aeruginosa* colonization	Yes	56 (58.3)	40 (41.7)	0.7188
					No	40 (55.6)	32 (44.4)	
INR	1.07 (1.00–1.13)	1.11 (1.04–1.18)	0.0178	Pancreatic sufficiency	Yes	21 (87.5)	3 (12.5)	0.0012
					No	75 (52.1)	69 (47.9)	
				Permanent inhaled antibiotics	Yes	27 (64.3)	15 (35.7)	0.2801
					No	69 (54.8)	57 (45.2)	
Enzyme dose [FIP/kg/day]	3554 (1992–5942)	3857 (2306–5284)	0.6189	Permanent oral antibiotics	Yes	31 (67.4)	15 (32.6)	0.0993
					No	65 (53.3)	57 (46.7)	
				Intravenous antibiotics (in last 3 months)	Yes	55 (55.0)	45 (45.0)	0.4961
					No	41 (60.3)	27 (39.7)	
Vitamin K dose [mg/kg/week]	0.40 (0.23–0.54)	0.28 (0.11–0.47)	0.0076	Oral antibiotics (in last 3 months)	Yes	22 (71.0)	9 (29.0)	0.0850
					No	74 (54.0)	63 (46.0)	
				Inhaled glucocorticoids therapy	Yes	41 (50.6)	40 (49.4)	0.0991
					No	55 (63.2)	32 (36.8)	
Vitamin K dose [mg/week]	10.3 (7.7–20.0)	8.4 (2.5–19.8)	0.0006	Vitamin K supplementation	<2.1 mg/week^2^	6 (27.3)	16 (72.7)	0.0024
					>2.1 mg/week	90 (61.6)	56 (38.4)	

^1^Severe class of mutations on both alleles of the *CFTR* gene.

^2^Patients not receiving vitamin K or receiving less than the recommended dosage for CF (0.3 mg/d).

**Table 3:** Clinical parameters in CF patients with normal and pathological u-OC percentage.

u-OC [%]										
Parameters Median (1^st^–3^rd^ quartile)	<20	20–50	≥50	p	Parameters (N%)		<20	20–50	≥50	p
Age [years]	12.1 (6.6–18.0)	19.1 (7.5–27.7)	14.7 (8.2–19.9)	0.0673	Age [years]	<18	78 (66.7)	10 (8.5)	29 (24.8)	0.0153
						≥18	26 (55.3)	12 (25.5)	9 (19.2)	
Body weight (Z-score)	−1.07 (−1.74– 0.42)	−1.33 (−1.86– −0.52)	−0.92 (−1.80– 0.40)	0.9044	Body weight (Z-score)	<−1	53 (64.6)	12 (14.6)	17 (20.8)	0.7216
						>−1	51 (62.2)	10 (12.2)	21 (25.6)	
Body height (Z-score)	−0.86 (−1.78– −0.22)	−0.47 (−2.08–0.13)	−0.74 (−2.10–0.09)	0.5186	Body height (Z-score)	<−1	45 (62.5)	10 (13.9)	17 (23.6)	0.9999
						>−1	59 (64.1)	12 (13.0)	21 (22.9)	
FEV1 [%]	72.6 (36.4–90.2)	40.2 (23.0–67.5)	58.0 (31.3–81.7)	0.0663	FEV1 [%]	<80%	51 (60.7)	14 (16.7)	19 (22.6)	0.2595
						>80%	29 (64.4)	3 (6.7)	13 (28.9)	
Albumin [g/dl]	3.87 (3.57–4.16)	3.81 (3.54–4.26)	3.90 (3.59–4.26)	0.3829	Albumin [g/dl]	<3.5	20 (71.4)	3 (10.7)	5 (17.9)	0.6250
						≥3.5	84 (61.8)	19 (14.0)	33 (24.2)	
ALT [U/l]	23.5 (17.0–31.0)	27.5 (18.0–43.5)	24.0 (20.0–37.0)	0.4220	*CFTR* gene mutation	F508del/F508del	45 (63.4)	8 (11.3)	18 (25.3)	0.6974
						other/other	59 (63.4)	14 (15.1)	20 (21.5)	
						s/s^1^	70 (68.0)	11 (10.7)	22 (21.3)	0.2235
						other/other	34 (55.7)	11 (18.0)	16 (26.3)	
AST [U/l]	28.0 (22.0–38.2)	33.0 (18.2–44.8)	28.0 (21.0–38.0)	0.9455	Liver diseases	Cirrhosis	5 (55.6)	1 (11.1)	3 (33.3)	0.6814
						Other liver involvement	30 (65.2)	8 (17.4)	8 (17.4)	
						No	69 (63.3)	13 (11.9)	27 (24.8)	
GGT [U/l]	13.0 (10.0–22.5)	20.0 (11.2–27.2)	19.0 (11.0–34.0)	0.1832	Diabetes	Yes	12 (66.7)	5 (27.8)	1 (5.5)	0.0502
						No	92 (63.0)	17 (11.6)	37 (25.4)	
					*Ps. aeruginosa* colonization	Yes	58 (60.4)	16 (16.7)	22 (22.9)	0.3346
						No	46 (67.6)	6 (8.8)	16 (23.6)	
INR	1.08 (1.01–1.15)	1.08 (0.98–1.15)	1.10 (1.01–1.16)	0.0943	Pancreatic sufficiency	Yes	12 (50.0)	5 (20.8)	7 (29.2)	0.2527
						No	92 (65.7)	17 (12.1)	31 (22.2)	
					Permanent inhaled antibiotics	Yes	29 (69.0)	5 (11.9)	8 (19.1)	0.7278
						No	75 (61.5)	17 (13.9)	30 (24.6)	
Enzyme dose [FIP/kg/day]	3698 (26656–5825)	2801 (1215–5162)	3309 (1773–5162)	0.2913	Permanent oral antibiotics	Yes	29 (64.4)	5 (11.1)	11 (24.5)	0.9338
						No	75 (63.0)	17 (14.3)	27 (22.7)	
					Intravenous antibiotics (in last 3 months)	Yes	64 (64.6)	17 (17.2)	18 (18.2)	0.0728
						No	40 (61.5)	5 (7.7)	20 (30.8)	
Vitamin K dose [mg/kg/week]	0.41 (0.25–0.61)	0.28 (0.19–0.49)	0.14 (0–0.37)	0.0001^2^	Oral antibiotics (in last 3 months)	Yes	20 (69.0)	4 (13.8)	5 (17.2)	0.7235
						No	84 (62.2)	18 (13.3)	33 (24.5)	
					Inhaled glucocorticoid therapy	Yes	53 (68.8)	9 (11.7)	15 (19.5)	0.3965
						No	51 (58.6)	13 (14.9)	23 (26.5)	
Vitamin K dose [mg/week]	12.0 (5.6–20.0)	10.0 (5.0–20.0)	6.7 (0–17.5)	0.0036^2^	Vitamin K supplementation	<2.1 mg/week^3^	5 (23.8)	3 (14.3)	13 (61.9)	<0.0001
						>2.1 mg/week	99 (69.2)	19 (13.3)	25 (17.5)	

^1^Severe class of mutations on both alleles of the *CFTR* gene

^2^u-OC < 20% vs 20–50% n.s; u-OC < 20 vs. >50% *p* < 0.05; u-OC 20–50% vs >50% n.s.

^3^Patients not receiving vitamin K or receiving less than the recommended dosage for CF (0.3 mg/d).

**Table 4:** Stepwise multiple regression analysis.

p model	Dependent variable	Independent variable	Beta	p
0.0032	PIVKA-II [ng/ml]	Liver disease	–1.710438	0.008
		Diabetes	–2.916903	0.025
		Glucocorticoid therapy	–1.673739	0.042
		*Ps. aeruginosa* colonization	1.471052	0.093
0.0026	u-OC [%]	Vitamin K dose [mg/kg/week]	–23.64407	0.005
		Glucocorticoid therapy	–9.665452	0.062
		Intravenous antibiotics (in last 3 months)	–9.377747	0.068
